# Are pre- and early pregnancy lifestyle factors associated with the risk of preterm birth? A secondary cohort analysis of the cluster-randomised GeliS trial

**DOI:** 10.1186/s12884-022-04513-5

**Published:** 2022-03-21

**Authors:** Roxana Raab, Julia Hoffmann, Monika Spies, Kristina Geyer, Dorothy Meyer, Julia Günther, Hans Hauner

**Affiliations:** 1 Institute of Nutritional Medicine, School of Medicine, Else Kröner-Fresenius-Centre for Nutritional Medicine, Technical University of Munich, Georg-Brauchle-Ring 62, 80992 Munich, Germany; 2 European Foundation for the Care of Newborn Infants, Hofmannstrasse 7a, 81379 Munich, Germany

**Keywords:** Preterm birth, Pregnancy, Healthy eating index, Antenatal lifestyle, Risk factors, Diet, Physical activity, Mental health

## Abstract

**Background:**

Maternal lifestyle is discussed as a modifiable determinant in the prevention of preterm birth. However, previous research on associations between individual lifestyle factors and preterm birth risk is inconclusive. In this secondary analysis, we investigated the associations between several modifiable antenatal lifestyle factors and the odds of preterm birth.

**Methods:**

This secondary cohort analysis used data from the cluster-randomised controlled “healthy living in pregnancy” (GeliS) trial. Data were collected from early pregnancy to birth with maternity records, validated questionnaires and birth protocols. Women with complete datasets for all covariates were eligible for analysis. Multivariate logistic regression models, adjusted for recognised risk factors, were fitted to determine whether dietary quality, assessed with a healthy eating index (HEI), physical activity (PA) levels and antenatal anxiety/distress influenced the odds of preterm birth. Moreover, the combined association between pre-pregnancy body mass index (BMI) and HEI on the odds of preterm birth was explored. The independent associations of individual dietary components and types of PA on prematurity were assessed by adjusted logistic regression models.

**Results:**

Overall, 1738 women were included in the analysis. A low HEI significantly increased the odds of preterm birth (OR 1.54 (CI 1.04 – 2.30), *p* = 0.033), while no associations with either low PA levels or antenatal anxiety/distress were observed. BMI significantly interacted with HEI on the association with prematurity (*p* = 0.036). Energy % from protein and the intake of average portions of vegetables and cereals were significantly negatively associated with the odds of preterm birth. There was no significant evidence of an association between different types of PA and prematurity.

**Conclusions:**

This cohort analysis revealed that low dietary quality in early pregnancy may increase the chance of giving birth prematurely, while healthier dietary choices may help to prevent preterm birth. More research on pre- and early pregnancy modifiable lifestyle factors is warranted.

**Trial registration:**

This trial is registered with the Clinical Trial Registry ClinicalTrials.gov (NCT01958307). Registration date 09 October 2013, retrospectively registered.

**Supplementary Information:**

The online version contains supplementary material available at 10.1186/s12884-022-04513-5.

## Background

Preterm birth is the leading cause of death in children under the age of five years [1]. Globally, around 15 million infants are born too soon [2]. The World Health Organization (WHO) defines prematurity as birth before 37 completed weeks of gestation [3]. Although the highest prevalence rates are observed in low- and middle-income countries, where adequate prenatal care is often lacking [2, 4], preterm birth remains a global health issue, with increasing rates in 62 out of 65 countries with reliable data [4]. In Germany, 8 – 9% of babies are born prematurely, which is one of the highest rates observed in Europe [5]. Preterm birth can affect the short-term health status of the newborn, including severe and life-threatening diseases such as necrotising enterocolitis, retinopathy, failure to thrive, metabolic disturbances and sepsis [3, 6]. It is also thought to influence the long-term health outcomes of both the infants and their families, with severe implications regarding their overall quality of life [3, 6]. In order to develop effective and efficacious prevention strategies there is an urgent need to elucidate the aetiology of preterm birth.

The causes of preterm birth are multifactorial and may be sociodemographic, biological, clinical, social-behavioural and environmental in nature [7, 8]. Although several risk factors have been discussed, including a history of preterm birth, infections, smoking during pregnancy, young and advanced age, extremes in body mass index (BMI) and socioeconomic disadvantages [7, 8], approximately two-thirds of preterm births in high-income countries occur for unknown reasons [9]. Thus, identification of further and, in particular, modifiable risk factors is urgently warranted. This has also been emphasised by global healthcare and parent organisations, such as the WHO, the Preterm Birth International Collaborative (PREBIC), and the European Foundation for the Care of Newborn Infants (EFCNI), who highlight the importance of maternal antenatal health and lifestyle parameters concerning preterm birth prevention [8, 10–12].

Previous studies have investigated potential associations between individual maternal lifestyle factors and the risk of preterm birth, including diet [13–22], physical activity (PA) [23, 24] and mental health [25–28]. Yet, findings have been inconclusive, and heterogeneity between studies made comparisons difficult. While some studies found that healthier dietary patterns [15, 20, 22], higher levels of PA [23, 24] and reduced levels of perceived stress [28, 29] lowered the risk of preterm birth, others could not confirm these associations [13, 17, 26, 30]. Given that the aetiology of preterm birth is multifactorial, factors may synergistically or antagonistically influence each other. It is therefore important to simultaneously consider the associations of potential modifiable and non-modifiable predictors on the odds of preterm birth. Moreover, most of the previous studies focused on factors in the second or third trimester [31, 32]. However, manifestation of beneficial lifestyle modifications is a matter of time, thus, identifying risk factors in pre- or early pregnancy may be of higher clinical relevance in terms of preterm birth prevention.

The objective of this cohort analysis was therefore to simultaneously investigate the associations between several pre- and early lifestyle risk factors, including dietary quality, PA levels and anxiety/distress, as well as putative socioeconomic and health risk factors on the odds of preterm birth. We additionally examined the potential influence of certain food groups and different types of PA on the odds of preterm birth.

## Methods

### Study design and participants

The cluster-randomised controlled “Gesund leben in der Schwangerschaft” (“healthy living in pregnancy”) (GeliS) trial was embedded in routine prenatal care and aimed at reducing the proportion of women with excessive gestational weight gain (GWG), as defined by the Institute of Medicine (IOM) [33]. Results on the primary and secondary endpoints have been reported elsewhere [34–40]. The study was conducted in accordance with the declaration of Helsinki and was approved by the ethics committee of the Technical University of Munich. A comprehensive description of the rationale, study design and methods is provided in the study protocol [33] and the trial register entry (ClinicalTrials.gov, NCT01958307).

In brief, participating midwifery and gynaecological practices from five regions in Bavaria, Germany, recruited pregnant women between 2013 and 2015. Women were eligible for participation if they met the following inclusion criteria: (1) pre-pregnancy BMI ≥ 18.5 kg/m^2^ and ≤ 40.0 kg/m^2^, (2) singleton pregnancy, (3) aged between 18 and 43 years, (4) ≤ 12 weeks of gestation, (5) sufficient German language skills, and (6) provision of written informed consent. Women were not eligible if they had multiple or complicated pregnancies or chronic diseases.

Women in the control group received standard prenatal care and a leaflet containing general information on healthy lifestyle and breastfeeding. Participants in the intervention group were offered three counselling sessions during pregnancy and one in the postpartum period alongside their routine care visits. Lifestyle counselling was given by previously trained midwives, gynaecologists or medical personnel and comprised personalised advice on recommended GWG, healthy diet, PA and breastfeeding [33].

### Data collection and study outcomes

#### Preterm and full-term birth

Data on gestational age at birth were derived from birth records. Preterm birth was defined as a live birth before 37 completed weeks of gestation, including both spontaneous and iatrogenic (induced labor or planned caesarean section) preterm birth. Preterm birth was further categorised into extremely, very, and moderate-to-late preterm born, referring to a gestational age of < 28 weeks, 28 – < 32 weeks, and 32 – < 37 weeks, respectively [3]. Infants born at 37 completed weeks of gestation or after were categorised as full-term birth.

#### Sociodemographic and clinical data

Maternal sociodemographic data were obtained by a screening questionnaire before the 12^th^ week of gestation. Educational level was classified as low when women graduated from general secondary school or lower.

Maternal pre-pregnancy weight was likewise collected via the screening questionnaire. The self-reported weight was used to categorise women into pre-pregnancy BMI classes (normal weight (BMI 18.5 – 24.9 kg/m^2^), overweight (BMI 25.0 – 29.9 kg/m^2^), and obesity (BMI 30.0 – 40.0 kg/m^2^)). Maternal weight during pregnancy was measured at each antenatal visit and recorded in maternity records. Early pregnancy GWG was calculated by subtracting the first measured weight in pregnancy (≤ 12 weeks of gestation) from the weight measured at the 16^th^ to 20^th^ week of gestation. Early GWG was further categorised into inadequate, adequate and excessive GWG according to the weekly GWG recommendations from the IOM [41]. It was assumed that women gain around 1 – 2 kg in the first trimester. Inadequate and excessive GWG were defined based on the lower (1 kg) and higher (2 kg) end of the range, respectively.

Between the 24^th^ and the 28^th^ week of gestation, women underwent a standardised 75 g oral glucose tolerance test (oGTT). According to national and international guidelines, women were diagnosed with gestational diabetes mellitus (GDM) if one or more of the following cut-off values was equalled or exceeded: Fasting plasma glucose: 92 mg/dL (5.1 mmol/L), 1 h: 180 mg/dL (10.0 mmol/L), and 2 h: 153 mg/dL (8.5 mmol/L) [42, 43].

#### Lifestyle factors

Women were asked to fill out a set of validated questionnaires in early pregnancy (≤ 12 weeks of gestation), covering pre- and early pregnancy lifestyle factors, including dietary and PA behaviour, smoking status, and mental health. Mothers who smoked during early pregnancy were classified as current smokers. Data on dietary intake over the last month were collected using the validated, and slightly modified, food frequency questionnaire (FFQ) originally developed by the Robert Koch Institute in Berlin, Germany, and applied in the German Health Interview and Examination Survey for Adults (DEGS) study [44]. This questionnaire enquired the consumption frequency and portion size of 54 different food items. Based on the collected dietary information, energy, macronutrient and fibre intake were estimated. Over- and underreporting of dietary behaviour was defined as previously reported [38], and women over- or underreporting dietary intake were excluded from analysis. Based on the FFQ, a healthy eating index (HEI) was calculated to estimate the dietary quality in accordance with the German national recommendations [45]. The score comprises values between 0 and 100. A score of 0 indicates the lowest and a score of 100 indicates the highest dietary quality. The median of women’s total HEI was used as a cut-off to categorise them as having a low or high HEI.

Data on PA behaviour were obtained using the Pregnancy Physical Activity Questionnaire (PPAQ), which has been slightly adapted to fit German habits [46]. The PPAQ comprises 32 questions related to the type and intensity of women’s PA behaviour during the last month. Based on the evaluation sheet of the PPAQ, time and intensity spent for each of the 32 activities was summed up to obtain the average weekly energy expenditure in metabolic equivalent of task (MET)-h/week [46]. Overreporting was defined as described elsewhere [35] and questionnaires of women overreporting their PA behaviour were excluded from analysis. MET-h/week of light intensities and above were summed up to total physical activity of light intensity and above (TALIA). The median of TALIA was used as cut-off to group women into high (above the median) or low (below the median) PA behaviour. Types of PA behaviour were derived from the PPAQ and included household, occupational, transportation, sports activity and inactivity.

Data on maternal mental health were collected using the German version of the Patient Health Questionnaire-4 (PHQ-4). A score of ≥ 3 on a scale of maximum 12 points indicates symptoms of anxiety and depression [47].

#### Statistical analysis

**Fig. 1:**
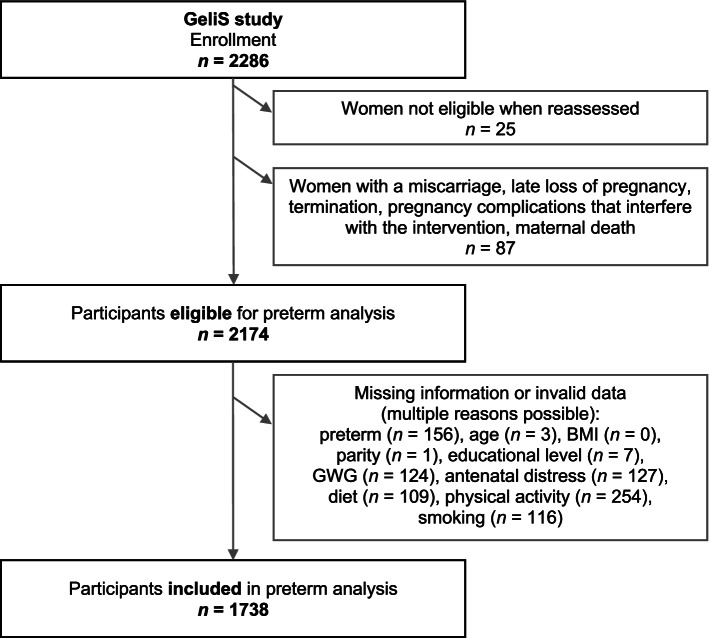
Flowchart of participants enrolled in the GeliS trial and included in preterm analysis. *BMI* body mass index, *GWG* gestational weight gain

Women with available information on preterm birth and complete data for all of the covariates listed above were included in the analysis. Participants who dropped out during pregnancy, as well as women with missing or invalid lifestyle data due to over- or underreporting, were excluded from analysis (see Fig. 1 for reasons of drop out). As the primary focus of this analysis was to identify antenatal predictors of preterm birth, and as there was no significant difference between intervention and control group in the incidence of preterm birth (Additional file 1: Table S1), data of both groups were pooled to form one cohort. In all analyses, group assignment was included as an adjustment factor to prevent potential confounding.

Baseline characteristics were stratified according to preterm birth status and are presented as means and standard deviations (SD) or frequencies and proportions. Differences between the groups were assessed by Chi-squared test for categorical and by Kruskal-Wallis test for continuous variables.

Multivariate logistic regression models were used to examine associations between potential predictors and the odds of preterm birth. Odds ratios (OR) with 95% confidence intervals (CI) were calculated. Four models with different sets of categorical and equivalent linear covariates were fitted. Model 1 included group assignment, maternal age, and pre-pregnancy BMI as covariates. Model 2 additionally assessed the influence of early inadequate or excessive GWG and nulliparity on the odds of preterm birth. Model 3 further comprised low education and smoking status. For the fully adjusted Model 4, low HEI, low PA and antenatal anxiety and distress were added. We further explored the potential combined association of HEI and BMI on the odds of preterm birth by including their interaction term into the fully adjusted categorical Model 4. Further logistic regression models were fitted to examine the influence of individual dietary and PA variables on the odds of preterm birth, adjusting for group assignment, maternal age, parity, and pre-pregnancy BMI.

Exploratory subgroup analyses of the multivariate logistic regression models were performed to assess the associations between the aforementioned covariates and the odds of spontaneous and iatrogenic preterm birth.

All analyses were performed using SPSS software (IBM SPSS Statistics for Windows, version 26.0, IBM Corp, Armonk, New York). A *p* value of < 0.05 was considered statistically significant. Due to the exploratory nature of this analysis, no adjustment for multiple comparisons was perfomed.

## Results

### Study sample and participant characteristics

Among 2286 enrolled women in the GeliS study, 2174 women were potentially eligible for the current analysis (Fig. 1). After exclusion of women with missing or implausible data for any of the covariates, the final analytical sample amounted to 1738 women. The characteristics of the original eligible and the finally included sample were comparable (Additional file 1: Table S2). The preterm birth rate did not differ between intervention and control groups (Additional file 1: Table S1).

Table 1 summarises maternal and infant characteristics of the included study population, stratified by preterm birth status. Overall, mothers were on average 30.4 ± 4.4 years old and had a mean pre-pregnancy BMI of 24.4 ± 4.5 kg/m^2^. Among the 1738 women, 114 (6.6%) had a preterm birth, of whom 100 (5.8%) were classified as moderate to late preterm, 12 (0.7%) as very preterm, and 2 (0.1%) as extremely preterm. Of the 112 women who gave birth to a preterm infant with additional information on the type of birth, 77 (68.8%) had a spontaneous and 35 (31.3%) had an iatrogenic preterm birth (Additional file 1: Table S3). Infants born preterm had lower birthweights as compared to infants born full-term (2370 ± 622 g vs. 3405 ± 436 g, *p* < 0.001) (Table 1). Mothers who gave birth to a preterm infant were more likely to be nulliparous compared to mothers who gave birth to a full-term infant (66.7% vs. 57.5%, *p* = 0.055). The proportion of mothers with a low HEI score was higher among those with a preterm birth, as compared to those with a full-term birth (59.6% vs. 49.3%, *p* = 0.033). All other sociodemographic, health and lifestyle factors were comparable between groups (Table 1).

### Associations between pre- and early pregnancy sociodemographic, health and lifestyle factors and the odds of preterm birth

Multivariate logistic regression analyses revealed significant associations between both nulliparity and advanced age (36 – 43 years) and the odds of preterm birth across Models 2 to 4 (Table 2). In the fully adjusted Model 4, low HEI was significantly positively associated with the odds of preterm birth (OR 1.54 (CI 1.04 – 2.30), *p* = 0.033). There was no significant evidence of an association between pre-pregnancy BMI, excessive or inadequate GWG, low PA, antenatal anxiety/distress, smoking or low education and the odds of preterm birth (Table 2).

**Table 1:** Characteristics of study participants with full-term vs. preterm birth

	**Full-term** (*n* = 1624, 93.4%)	**Preterm** (*n* = 114, 6.6%)	**Total** (*n* = 1738)	***p* value**^a^
***Maternal characteristics***				
** Group allocation**^b^				
Control group	792/1624 (48.8%)	50/114 (43.9%)	842/1738 (48.4%)	0.311
Intervention group	832/1624 (51.2%)	64/114 (56.1%)	896/1738 (51.6%)	
** Pre-pregnancy age (years)**^c^	30.3 ± 4.4	31.0 ± 4.4	30.4 ± 4.4	0.139
** Pre-pregnancy weight (kg)**	68.2 ± 13.5	67.9 ± 13.2	68.2 ± 13.4	0.906
** Pre-pregnancy BMI (kg/m**^**2**^**)**	24.3 ± 4.5	24.6 ± 4.5	24.4 ± 4.5	0.479
** Pre-pregnancy BMI category (*n* (%))**				
BMI 18.5 – 24.9 kg/m^2^	1069/1624 (65.8%)	68/114 (59.6%)	1137/1738 (65.4%)	0.332
BMI 25.0 – 29.9 kg/m^2^	362/1624 (22.3%)	32/114 (28.1%)	394/1738 (22.7%)	
BMI 30.0 – 40.0 kg/m^2^	193/1624 (11.9%)	14/114 (12.3%)	207/1738 (11.9%)	
** Early GWG (kg)**^d^	2.8 ± 2.4	2.6 ± 2.2	2.8 ± 2.4	0.381
** Early GWG category (*n* (%))**^e^				
Inadequate	432/1624 (26.6%)	34/114 (29.8%)	466/1738 (26.8%)	0.754
Adequate	196/1624 (12.1%)	13/114 (11.4%)	209/1738 (12.0%)	
Excessive	996/1624 (61.3%)	67/114 (58.8%)	1063/1738 (61.2%)	
** GDM (*n* (%))**^f^	166/1566 (10.6%)	14/109 (12.8%)	180/1675 (10.7%)	0.465
** Educational level (*n* (%))**				
General secondary school^g^	227/1624 (14.0%)	18/114 (15.8%)	245/1738 (14.1%)	0.835
Vocational secondary school	704/1624 (43.3%)	47/114 (41.2%)	751/1738 (43.2%)	
Academic high school	693/1624 (42.7%)	49/114 (43.0%)	742/1738 (42.7%)	
** Country of birth (*n* (%))**				
Germany	1459/1622 (90.0%)	97/114 (85.1%)	1556/1736 (89.6%)	0.100
Other	163/1622 (10.0%)	17/114 (14.9%)	180/1736 (10.4%)	
** Native language (*n* (%))**				
German	1537/1622 (94.8%)	109/114 (95.6%)	1646/1736 (94.8%)	0.691
Other	85/1622 (5.2%)	5/114 (4.4%)	90/1736 (5.2%)	
** Nulliparous (*n* (%))**	934/1624 (57.5%)	76/114 (66.7%)	1010/1738 (58.1%)	0.055
** Living with a partner (*n* (%))**	1564/1619 (96.6%)	111/114 (97.4%)	1675/1733 (96.7%)	0.660
** Married (*n* (%))**	1077/1618 (66.6%)	70/114 (61,4%)	1147/1732 (66.2%)	0.260
** Full-time employed (*n* (%))**	857/1612 (53.2%)	69/113 (61.1%)	926/1725 (53.7%)	0.104
** Current smoker (*n* (%))**	81/1624 (5.0%)	4/114 (3.5%)	85/1738 (4.9%)	0.479
** Low HEI (*n* (%))**^h^	801/1624 (49.3%)	68/114 (59.6%)	869/1738 (50.0%)	**0.033**
** Low PA (*n* (%))**^i^	807/1624 (49.7%)	62/114 (54.4%)	869/1738 (50.0%)	0.333
** Antenatal distress (*n* (%))**^j^	682/1624 (42.0%)	48/114 (42.1%)	730/1738 (42.0%)	0.982
** Low well-being (*n* (%))**^k^	589/1609 (36.6%)	38/110 (34.5%)	627/1719 (36.5%)	0.664
***Infant characteristics***				
** Infant sex (*n* (%))**				
Male	825/1608 (51.3%)	62/110 (56.4%)	887/1718 (51.6%)	0.304
Female	783/1608 (48.7%)	48/110 (43.6%)	831/1718 (48.4%)	
** Birthweight (g)**	3405 ± 436	2370 ± 622	3337 ± 518	**< 0.001**

*BMI* body mass index, *GDM* gestational diabetes mellitus, *GWG* gestational weight gain, *HEI* Healthy Eating Index, *IOM* Institute of Medicine, *oGTT* oral glucose tolerance test, *PA* physical activity, *PHQ-4* Patient Health Questionnaire-4, *SD* standard deviation, *TALIA* total physical activity of light intensity and above, *WHO-5* World Health Organization Well-Being Index 5

^a^*p* value for differences between women with full-term and preterm birth, tested with χ^2^ test for categorical variables and Kruskal-Wallis test for continuous variables

^b^Frequency (percent) (all such values)

^c^Mean ± SD (all such values)

^d^GWG until the 16^th^ – 20^th^ week of gestation

^e^Defined according to the criteria of the IOM

^f^Assessed by an 75 g oGTT in the 24^th^ – 28^th^ week of gestation

^g^General secondary school, which is completed through year 9

^h^HEI below the median of the analysed population

^i^TALIA below the median of the analysed population

^j^PHQ-4 score of ≥ 3 points

^k^WHO-5 score of < 50%

In an exploratory analysis a significant interactive association between pre-pregnancy BMI and HEI on the odds of preterm birth was observed (*p* = 0.036, data not shown).

Multivariate analyses were also performed with the inclusion of the same predictor variables, noted above, as continuous variables. Age was significantly positively associated with the odds of preterm birth across Models 2 to 4 (Model 4: OR 1.05 (1.00 – 1.10), *p* = 0.037) (Additional file 1: Table S4). None of the other factors were significantly linked to the odds of preterm birth in the linear models.

Overall, the Akaike Information Criterion (AIC) and Bayesian Information Criterion (BIC) were lower across categorical as compared to linear models (data not shown). Lower information criteria indicate a better fit of the model [48].

Subgroup analyses exploring possible associations of the included covariates with the type of preterm birth revealed advanced age to be significantly positively associated with the odds of iatrogenic preterm birth across categorical Models 2 to 4 (Model 4: OR 5.99 (1.20 – 29.79), *p* = 0.029) and across linear Models 1 to 4 (Model 4: OR 1.12 (1.04 – 1.22), *p* = 0.004) (Additional File 1: Table S5 and Table S6). Nulliparity was significantly positively associated with the odds of spontaneous preterm birth across categorical Models 2 to 3 (Model 3: OR 1.69 (1.02 – 2.80), *p* = 0.043) (Additional file 1: Table S7 and Table S8).

### Associations between specific dietary and physical activity variables and the odds of preterm birth

Table 3 depicts results from regression analyses investigating whether the intake of certain macronutrients, food groups and different types of PA are associated with the odds of preterm birth. There was no evidence of an association between overall energy intake and the odds of preterm birth, while energy % (E%) from certain macronutrients and food groups was associated with prematurity. The odds of preterm birth were significantly decreased by 53% per 10 E% derived from protein (*p* = 0.030). E% from both carbohydrates in general and from saccharose tended to increase the odds of preterm birth, without evidence of a robust association (*p* = 0.071 and *p* = 0.062, respectively).

**Table 2:** Associations between sociodemographic, health and lifestyle factors and the odds of preterm birth

Covariate	Model 1	Model 2	Model 3	Model 4
**Group assignment**	1.20 (0.82 – 1.76)	1.14 (0.78 – 1.68)	1.15 (0.78 – 1.69)	1.18 (0.80 – 1.73)
**BMI category**^a^				
BMI 25.0 – 29.9 kg/m^2^	1.39 (0.89 – 2.15)	1.43 (0.92 – 2.22)	1.41 (0.90 – 2.20)	1.37 (0.88 – 2.14)
BMI 30.0 – 40.0 kg/m^2^	1.14 (0.63 – 2.08)	1.10 (0.60 – 2.02)	1.09 (0.59 – 1.99)	1.04 (0.56 – 1.91)
**Age**^b^				
26 – 35 years	1.52 (0.78 – 2.98)	1.69 (0.86 – 3.32)	1.70 (0.86 – 3.36)	1.82 (0.92 – 3.63)
36 – 43 years	2.06 (0.94 – 4.55)	**2.52 (1.12 – 5.65)**^**+**^	**2.53 (1.13 – 5.67)**^**+**^	**2.70 (1.20 – 6.10)**^**+**^
**Nulliparity**		**1.63 (1.08 – 2.45)**^**+**^	**1.64 (1.08 – 2.47)**^**+**^	**1.58 (1.02 – 2.42)**^**+**^
**Early GWG**^c^				
Excessive		0.99 (0.53 – 1.84)	1.00 (0.54 – 1.86)	0.98 (0.53 – 1.83)
Inadequate		1.23 (0.63 – 2.41)	1.25 (0.64 – 2.44)	1.18 (0.60 – 2.32)
**Smoking**^d^			0.70 (0.25 – 1.97)	0.64 (0.23 – 1.81)
**Low education**^e^			1.25 (0.73 – 2.14)	1.18 (0.68 – 2.03)
**Low HEI**^f^				**1.54 (1.04 – 2.30)**^**+**^
**Low PA**^g^				1.04 (0.69 – 1.55)
**Antenatal anxiety/** **distress**^h^				1.01 (0.69 – 1.50)

*BMI* body mass index, *GWG* gestational weight gain, *HEI* Healthy Eating Index, *IOM* Institute of Medicine, *PA* physical activity, *PHQ-4* Patient Health Questionnaire-4, *TALIA* total physical activity of light intensity and above

^+^ *p* < 0.05

^a^BMI 18.5 – 24.9 kg/m^2^ was used as reference

^b^Age 18 – 25 years was used as reference

^c^GWG until the 16^th^ – 20^th^ week of gestation, defined according to the criteria of the IOM, adequate GWG was used as reference

^d^Current smoker

^e^General secondary school or lower

^f^HEI below the median of the analysed population

^g^TALIA below the median of the analysed population

^h^PHQ-4 score of ≥ 3 points

**Table 3:** Associations between specific dietary and physical activity variables and the odds of preterm birth

	***n***	**OR (CI)**	***p* value**^a^
**Energy and macronutrient intake**			
Energy intake (per 100 kcal/day)	1584	1.00 (0.97 – 1.04)	0.940
E% fat (per 10 E%/day)	1584	0.80 (0.57 – 1.11)	0.175
E% saturated fat (per 10 E%/day)	1584	0.74 (0.40 – 1.38)	0.345
E% protein (per 10 E%/day)	1584	0.47 (0.24 – 0.93)	**0.030**
E% carbohydrates (per 10 E%/day)	1584	1.27 (0.98 – 1.64)	0.071
Saccharose (per 10 g/day)	1584	1.06 (0.98 – 1.12)	0.062
Fibre (per 10 g/day)	1584	0.93 (0.76 – 1.15)	0.511
Alcohol (per g/day)	1584	1.06 (0.99 – 1.14)	0.097
Caffeine (100 mg/day)	1582	1.05 (0.82 – 1.33)	0.710
**Food intake**			
Soft drinks (200 ml/day)	1737	1.01 (0.95 – 1.07)	0.843
Sweets and snacks (50 g/day)	1738	0.95 (0.79 – 1.15)	0.628
Fast food (250 g/day)	1737	1.04 (0.27 – 4.00)	0.961
Meat and meat products (150 g/day)	1737	0.79 (0.45 – 1.38)	0.408
Fish (90 g/day)	1737	0.61 (0.14 – 2.62)	0.508
Dairy products (200 g/day)	1738	0.86 (0.73 – 1.02)	0.078
Cheese (30 g/day)	1734	0.82 (0.64 – 1.05)	0.108
Eggs (60 g/day)	1727	0.49 (0.19 – 1.26)	0.138
Cereals (50 g/day)	1738	0.71 (0.59 – 0.85)	** < 0.001**
Nuts (25 g/day)	1734	0.54 (0.16 – 1.80)	0.313
Fruits (150 g/day)	1737	1.05 (0.97 – 1.14)	0.234
Vegetables (150 g/day)	1736	0.75 (0.59 – 0.96)	**0.023**
Vegetarian	1727	0.66 (0.26 – 1.65)	0.372
**Physical activity**^b^			
Total PA	1738	1.02 (0.70 – 1.49)	0.924
Household PA	1738	0.98 (0.94 – 1.02)	0.333
Occupational PA	1330	1.00 (0.99 – 1.00)	0.694
Sports	1738	0.92 (0.74 – 1.14)	0.447
Transportation PA	1738	0.94 (0.80 – 1.11)	0.462
Inactivity	1728	1.07 (0.94 – 1.23)	0.287

*CI* confidence interval, *E%* energy percent, *MET* metabolic equivalent of task, *OR* odds ratio, *PA* physical activity

^a^Adjusted for maternal pre-pregnancy BMI, age, parity and group assignment

^b^Effect sizes are calculated per 10 MET-h/week

Among food groups, vegetable intake and cereal intake, per average portion, lowered the odds of preterm birth by 25% (*p* = 0.023) and by 29% (*p* < 0.001), respectively. Consumption of dairy products yielded a similar trend (*p* = 0.078). No further food groups were significantly associated with the odds of preterm birth (Table 3).

There was no evidence of associations for total PA or any of the different types of PA, including household, occupational, sports, transportation or inactivity with the odds of giving birth to a preterm infant (Table 3).

## Discussion

This secondary analysis of pooled data from the GeliS cohort investigated the simultaneous associations between several modifiable and non-modifiable pre- and early pregnancy, sociodemographic, health and lifestyle factors and the risk of preterm birth. The findings suggest that women with a low HEI in early pregnancy have a higher risk of giving birth to a preterm infant. An exploratory analysis further elucidated that there was a significant combined association between low dietary quality and higher pre-pregnancy BMI on the risk of preterm birth. More specific associations between selected dietary factors and preterm birth risk revealed that E% from protein and the intake of vegetables and cereals reduced the odds. None of the other modifiable risk factors, including low PA and anxiety or distress in pre- and early pregnancy, were significantly associated with the risk of prematurity.

The causes of prematurity are still largely unknown, which makes the identification of early risk factors highly relevant for clinical practice. Within the last few decades, research has focused on identifying maternal sociodemographic and health characteristics as risk factors for preterm birth. Our data confirmed that advanced age and nulliparity increase the risk for preterm birth, as already noted previously [49]. Subgroup analyses by the type of preterm birth revealed advanced age to increase the risk for iatrogenic and nulliparity to increase the risk for spontaneous preterm birth, which is in line with previous investigations [49–51]. While previous research suggests that extremes in BMI, including underweight and overweight/obesity, and inadequate as well as excessive GWG are risk factors for preterm birth [52–54], we found no evidence of independent associations in our multivariate models. In our analyses, neither smoking nor low education showed an influence on preterm birth risk. Although our findings contradict previous research observing associations with these maternal factors and preterm birth [7, 55], differences might be explained by the relatively high educational level and the rather low rate of smokers in the GeliS cohort [56].

Besides maternal sociodemographic and health characteristics, lifestyle factors are discussed to play a crucial role in preventing preterm birth. So far, published research focused on analysing lifestyle factors independently. However, factors might interact with each other, which emphasizes to explore the combined associations of predictors on preterm birth risk.

Recent observations of lower preterm birth rates during the lockdown phases of the ongoing COVID-19 pandemic suggest that drastic changes to women’s lifestyles may emerge as possible contributory factors [57–61]. Besides changes in dietary and PA habits, one discussed factor is stress, and in particular work related stress, which might have been reduced during the pandemic [57, 61]. In agreement with this hypothesis, a recent cohort analysis reported that perceived stress significantly increased the risk for preterm birth [29]. However, other mental health outcomes, including antenatal depressive symptoms, were not found to be associated with preterm birth risk in this cohort [29], which is in line with our observations. More research is needed to clarify the role of women’s mental health with regard to preterm birth risk.

In addition to maternal mental health, diet and PA have been discussed to influence preterm birth risk. In line with our findings, recent systematic reviews [31, 62, 63] have found that healthier dietary patterns, characterised by a high intake of vegetables, fruits and whole grains, reduce the risk of preterm birth, while unhealthier western-type dietary patterns had the opposite effect [64]. Notably, studies investigating whether certain diets influence the risk for preterm birth were inconsistent. For instance, adherence to a Mediterranean diet has been linked to reduced preterm birth risk in a Danish cohort [20], while no association was observed in a Norwegian cohort [17]. In the Norwegian MoBa cohort, adherence to the new Nordic diet lowered the risk for preterm birth [18]. In two cohorts from Asia and Australia, overall dietary quality assessed by a HEI had no influence on overall preterm birth risk [13, 15], although the Australian study found higher dietary quality to reduce the risk of preterm birth among women with obesity [15]. A similar observation was made by Saunders et al. [19]. In an exploratory analysis, we confirmed pre-pregnancy BMI to significantly interact with HEI on the association with preterm birth risk.

Inconsistencies in findings related to dietary parameters and their contribution to the risk of preterm birth may be attributable to differences in dietary assessment methods, timing of assessment, methodological approaches and heterogeneity in population characteristics, which makes a general comparison between study results difficult.

The potential of PA as another modifiable risk factor for preterm birth risk is not yet fully understood. We did not observe any association between low PA and the odds of preterm birth in the combined model estimate. While some of the previous studies explicitly described leisure time PA/overall PA to reduce preterm birth risk [32, 65], others observed no influence [66, 67]. Our research group previously investigated the impact of different PA intensities on preterm birth risk [68]. Sedentary behaviour tended to increase the risk of preterm birth, as did vigorous PA behaviour in late pregnancy. In line with these observations, other studies have observed a U-shaped trend between PA intensity/amount and preterm birth risk, indicating that light-to-moderate PA seems to be safe and should be aspired to, as its beneficial effects extend beyond risk reduction and enhance a woman’s well-being [23, 69]. Different from our previous investigation on PA intensity [68], the present analysis endeavoured to assess the link between the less investigated types/purposes of PA, including household, transportation, occupation, sports and inactivity and the risk for preterm birth. None of these PA types were associated with prematurity, which is in line with previous literature [32, 65]. These findings lead to the suggestion that not the type of PA per se, but rather PA intensity and potentially also duration seems to confer risk lowering effects on preterm birth.

### Limitations

Our findings should be interpreted in light of some limitations. Despite using validated questionnaires to assess maternal lifestyle, self-reported data could have introduced reporting bias. Although we used a validated FFQ, we acknowledge that the calculated E% is only an estimate and may not be completely accurate. Although we adjusted for a variety of non-modifiable and modifiable covariates in our multivariate models, residual confounding by other risk factors, such as ethnicity or a history of preterm birth, cannot be excluded. We are aware that some of the reported OR for the influence of specific food groups are rather small and their clinical relevance has to be interpreted with caution. As the data are taken from a well-educated sample of women from a single federal state, generalising our findings to the broader German population may be limited. The lack of more detailed information on the ethnicity of the study participants is a clear limitation which needs to be acknoweledged. The exclusion of women with complicated or multiple pregnancies, which are known to have an increased risk for preterm birth [7], could explain the lower incidence of preterm births in our cohort compared to the figures for Germany [5]. However, in these women, factors other than lifestyle may play a major role in increasing the risk of preterm birth, making them less relevant to this analysis.

### Strengths

This comprehensive analysis has several strengths that merit particular attention. This study adds to the evidence the simultaneous consideration of modifiable and non-modifiable maternal determinants in pre- and early pregnancy on the odds of preterm birth. Moreover, few research groups have examined so far whether pre- and early pregnancy lifestyle factors play a role in increasing the risk for preterm birth. Dietary quality was rated with a HEI. This tool might be advantageous over previous approaches, such as dietary pattern analyses, as it was based on national recommendations. The GeliS study considered women of several BMI classes, which enabled us to identify a combined association between pre-pregnancy BMI and HEI on the odds of prematurity. Our study was conducted within the German routine care system and thus allowed the collection of data from medical records with minimal inconvenience to the participants and, consequently, low drop-out rates.

## Conclusions

Preterm birth is a global health issue, for which the causes are still largely unknown. Our findings suggest that a low dietary quality is associated with an increased risk of preterm birth, while a healthier diet may contribute to prevent prematurity. Further research on early modifiable risk factors is needed to develop prevention strategies. Cohort data allowing a pre- and within-pandemic comparison may be additionally valuable for further elucidation.

## Supplementary Information

**Additional file 1:** **Table S1 **Preterm incidence in intervention and control group. **Table S2 **Characteristics of eligible and included participants. **Table S3 **Proportions of spontaneous and iatrogenic preterm births. **Table S4 **Associations between sociodemographic, health and lifestyle factors and the odds of preterm birth – models including linear covariates. **Table S5 **Associations between sociodemographic, health and lifestyle factors and the odds of iatrogenic preterm birth – models including categorical covariates. **Table S6 **Associations between sociodemographic, health and lifestyle factors and the odds of iatrogenic preterm birth – models including linear covariates. **Table S7 **Associations between sociodemographic, health and lifestyle factors and the odds of spontaneous preterm birth – models including categorical covariates. **Table S8 **Associations between sociodemographic, health and lifestyle factors and the odds of spontaneous preterm birth – models including linear covariates. 

## Data Availability

The datasets generated and/or analysed during the current study are not publicly available due to data privacy/legal restrictions but are available from the corresponding author on reasonable request.

## Abbreviations

WHO: World Health Organization
BMI: Body Mass Index
PREBIC: Preterm Birth International Collaborative
EFCNI: European Foundation for the Care of Newborn Infants
PA: Physical Activity
GeliS: Gesund leben in der Schwangerschaft (“healthy living in pregnancy”)
GWG: Gestational Weight Gain
IOM: Institute of Medicine
oGTT: Oral Glucose Tolerance Test
GDM: Gestational Diabetes Mellitus
FFQ: Food Frequency Questionnaire
DEGS: German Health Interview and Examination Survey for Adults
HEI: Healthy Eating Index
PPAQ: Pregnancy Physical Activity Questionnaire
MET: Metabolic Equivalent of Task
TALIA: Total Physical Activity of Light Intensity and Above
PHQ-4: Patient Health Questionnaire-4
SD: Standard Deviation
OR: Odds Ratio
CI: Confidence Interval
AIC: Akaike Information Criterion
BIC: Bayesian Information Criterion
