# Supplementary material for: Are pre- and early pregnancy lifestyle factors associated with the risk of preterm birth? A secondary cohort analysis of the cluster-randomised GeliS trial
Source: BMC Pregnancy Childbirth. 2022 Mar 21;22:230. doi: 10.1186/s12884-022-04513-5 (PMC8935257; doi:10.1186/s12884-022-04513-5)
Supplement: Supplementary file 1 — Additional file 1: Table S1 Preterm incidence in intervention and control group. Table S2 Characteristics of eligible and included participants. Table S3 Proportions of spontaneous and iatrogenic preterm births. Table S4 Associations between sociodemographic, health and lifestyle factors and the odds of preterm birth – models including linear covariates. Table S5 Associations between sociodemographic, health and lifestyle factors and the odds of iatrogenic preterm birth – models including categorical covariates. Table S6 Associations between sociodemographic, health and lifestyle factors and the odds of iatrogenic preterm birth – models including linear covariates. Table S7 Associations between sociodemographic, health and lifestyle factors and the odds of spontaneous preterm birth – models including categorical covariates. Table S8 Associations between sociodemographic, health and lifestyle factors and the odds of spontaneous preterm birth – models including linear covariates. [file 12884_2022_4513_MOESM1_ESM.pdf]

Additional File 1

Table S1 Preterm incidence in intervention and control group

Preterm status	Intervention *n* (%)	Control *n* (%)	Unadjusted OR (95% CI)	Adjusted OR (95% CI)[a]
**Preterm**	832/896 (92.9%)	792/842 (94.1%)	1.22 (0.83 – 1.79)	1.17 (0.80 – 1.73)
**Full-term**	64/896 (7.1%)	50/842 (5.9%)		

BMI body mass index, *OR* odds ratio

[a] adjusted for maternal pre-pregnancy BMI, age, parity

Table S2 Characteristics of eligible and included participants

	**Eligible**[a] (*n* = 2174)	**Included**[b] (*n* = 1738)
Maternal characteristics		
**Group allocation**[c]		
Control group	1075/2174 (49.4%)	842/1738 (48.4%)
Intervention group	1099/2174 (50.6%)	896/1738 (51.6%)
**Pre-pregnancy age (years)**[d]	30.2 ± 4.5	30.4 ± 4.4
**Pre-pregnancy weight (kg)**	68.2 ± 13.4	68.2 ± 13.4
**Pre-pregnancy BMI (kg/m^2)**	24.4 ± 4.5	24.4 ± 4.5
**Pre-pregnancy BMI category (*n* (%))**		
BMI 18.5 – 24.9 kg/m^2	1412/2174 (64.9%)	1137/1738 (65.4%)
BMI 25.0 – 29.9 kg/m^2	500/2174 (23.0%)	394/1738 (22.7%)
BMI 30.0 – 40.0 kg/m^2	262/2174 (12.1%)	207/1738 (11.9%)
**Early GWG (kg)**[e]	2.8 ± 2.42	2.8 ± 2.40
**Early GWG category (*n* (%))**[f]		
Inadequate	543/2050 (26.5%)	466/1738 (26.8%)
Adequate	249/2050 (12.1%)	209/1738 (12.0%)
Excessive	1258/2050 (61.4%)	1063/1738 (61.2%)
**GDM (*n* (%))**[g]	215/1968 (10.9%)	180/1675 (10.7%)
**Educational level (*n* (%))**		
General secondary school[h]	347/2167 (16.0%)	245/1738 (14.1%)
Vocational secondary school	913/2167 (42.1%)	751/1738 (43.2%)
Academic high school	907/2167 (41.9%)	742/1738 (42.7%)
**Country of birth (*n* (%))**		
Germany	1923/2169 (88.7%)	1556/1736 (89.6%)
Other	246/2169 (11.3%)	180/1736 (10.4%)
**Native language (*n* (%))**		
German	2037/2169 (93.9%)	1646/1736 (94.8%)
Other	132/2169 (6.1%)	90/1736 (5.2%)
**Nulliparous (*n* (%))**	1252/2173 (57.6%)	1010/1738 (58.1%)

Table S2 Continued

	**Eligible**[a] (*n* = 2174)	**Included**[b] (*n* = 1738)
**Living with a partner (*n* (%))**	2075/2166 (95.8%)	1675/1733 (96.7%)
**Married (*n* (%))**	1399/2163 (64.7%)	1147/1732 (66.2%)
**Full-time employed (*n* (%))**	1111/2149 (51.7%)	926/1725 (53.7%)
**Current smoker (*n* (%))**	121/2058 (5.9%)	85/1738 (4.9%)
**Low HEI (*n* (%))**[i]	1010/2005 (50.4%)	870/1738 (50.1%)
**Low PA (*n* (%))**[j]	961/1920 (50.1%)	882/1738 (50.7%)
**Antenatal distress (*n* (%))**[k]	872/2047 (42.6%)	730/1738 (42.0%)
**Low well-being (*n* (%))**[l]	744/2037 (36.5%)	627/1719 (36.5%)
Infant characteristics		
**Infant sex (*n* (%))**		
Male	1039/1996 (52.1%)	887/1718 (51.6%)
Female	957/1996 (47.9%)	831/1718 (48.4%)
**Birthweight (g)**	3338 ± 518	3337 ± 518

BMI body mass index, *GDM* gestational diabetes mellitus, *GWG* gestational weight gain, *HEI* Healthy Eating Index, *IOM* Institute of Medicine, *oGTT* oral glucose tolerance test, *PA* physical activity, *PHQ-4* Patient Health Questionnaire-4, *SD* standard deviation, *TALIA* total physical activity of light intensity and above, *WHO-5* World Health Organization Well-Being Index 5

[a] Participants initially eligible for preterm analysis

[b] Participants finally included in preterm analysis (refers to the group of total participants in Table 1 of the main manuscript)

[c] Frequency (percent) (all such values)

[d] Mean ± SD (all such values)

[e] GWG until the 16th – 20th week of gestation

[f] Defined according to the criteria of the IOM

[g] Assessed by an 75 g oGTT in the 24th – 28th week of gestation

[h] General secondary school, which is completed through year 9

[i] HEI below the median of the analysed population

[j] TALIA below the median of the analysed population

[k] PHQ-4 score of ≥ 3 points

[l] WHO-5 score of < 50%

Table S3 Proportions of spontaneous and iatrogenic preterm births

**Type of preterm birth**	***n* (%)**[a]
**Spontaneous**	77/112 (68.8%)
**Iatrogenic**	35/112 (31.3%)

[a] including women with available information on type of birth

Table S4 Associations between sociodemographic, health and lifestyle factors and the odds of preterm birth – models including linear covariates

Covariate	Model 1	Model 2	Model 3	Model 4
**Group assignment**	1.23 (0.84 – 1.80)	1.18 (0.80 – 1.73)	1.19 (0.81 – 1.75)	1.20 (0.82 – 1.77)
**BMI**[a]	1.01 (0.97 – 1.05)	1.01 (0.96 – 1.05)	1.00 (0.96 – 1.05)	1.00 (0.96 – 1.05)
**Age**	1.03 (0.99 – 1.08)	**1.05 (1.00 – 1.10)**[+]	**1.05 (1.00 – 1.10)**[+]	**1.05 (1.00 – 1.10)**[+]
**Parity**		0.77 (0.57 – 1.05)	0.77 (0.56 – 1.05)	0.82 (0.59 – 1.12)
**Early GWG**[b]		0.96 (0.88 – 1.04)	0.96 (0.88 – 1.04)	0.96 (0.89 – 1.05)
**Smoking**[c] **(categorial)**			0.73 (0.26 – 2.05)	0.68 (0.24 – 1.93)
**Low education**[d] **(categorial)**			1.28 (0.75 – 2.20)	1.25 (0.73 – 2.15)
**HEI score**[e]				0.86 (0.69 – 1.09)
**PA**[f]				0.98 (0.95 – 1.01)
**Antenatal distress**[g] **(categorial)**				1.01 (0.69 – 1.50)

BMI body mass index, *GWG* gestational weight gain, *HEI* Healthy Eating Index, *MET* metabolic equivalent of task, *PA* physical activity, *PHQ-4* Patient Health Questionnaire-4, *TALIA* total physical activity of light intensity and above

[+] $p < 0.05$

[a] Effect sizes are calculated per unit of measurement respectively

[b] GWG until the 16th – 20th week of gestation

[c] Current smoker

[d] General secondary school or lower

[e] Effect sizes are calculated per 10 HEI points

[f] TALIA, effect sizes are calculated per 10 MET-h/week

[g] PHQ-4 score of ≥ 3 points

Table S5 Associations between sociodemographic, health and lifestyle factors and the odds of iatrogenic preterm birth – models including categorical covariates

Covariate	Model 1	Model 2	Model 3	Model 4
**Group assignment**	0.61 (0.31 – 1.20)	0.59 (0.29 – 1.17)	0.58 (0.29 – 1.16)	0.59 (0.30 – 1.18)
**BMI category**[a]				
BMI 25.0 – 29.9 kg/m^2	1.64 (0.78 – 3.46)	1.62 (0.76 – 3.43)	1.60 (0.75 – 3.40)	1.55 (0.73 – 3.31)
BMI 30.0 – 40.0 kg/m^2	1.06 (0.36 – 3.15)	0.99 (0.33 – 2.98)	0.99 (0.33 – 2.98)	0.94 (0.31 – 2.87)
**Age**[b]				
26 – 35 years	2.29 (0.54 – 9.75)	2.56 (0.60 – 11.00)	2.66 (0.61 – 11.53)	2.89 (0.66 – 12.63)
36 – 43 years	4.38 (0.92 – 20.88)	**5.35 (1.09 – 26.19)**[+]	**5.53 (1.13 – 27.21)**[+]	**5.99 (1.20 – 29.79)**[+]
**Nulliparity**		1.56 (0.77 – 3.18)	1.59 (0.78 – 3.24)	1.51 (0.72 – 3.18)
**Early GWG**[c]				
Excessive		2.00 (0.46 – 8.54)	1.93 (0.45 – 8.36)	1.88 (0.44 – 8.15)
Inadequate		2.55 (0.55 – 11.76)	2.50 (0.54 – 11.54)	2.38 (0.51 – 11.02)
**Smoking**[d]			1.99 (0.57 – 6.89)	1.79 (0.51 – 6.29)
**Low education**[e]			0.94 (0.35 – 2.53)	0.88 (0.32 – 2.41)
**Low HEI**[f]				1.46 (0.72 – 2.96)
**Low PA**[g]				1.02 (0.50 – 2.07)
**Antenatal anxiety/ distress**[h]				1.17 (0.59 – 2.32)

BMI body mass index, *GWG* gestational weight gain, *HEI* Healthy Eating Index, *IOM* Institute of Medicine, *PA* physical activity, *PHQ-4* Patient Health Questionnaire-4, *TALIA* total physical activity of light intensity and above

[+] $p < 0.05$

[a] BMI 18.5 – 24.9 kg/m^2 was used as reference

[b] Age 18 – 25 years was used as reference

[c] GWG until the 16th – 20th week of gestation, defined according to the criteria of the IOM, adequate GWG was used as reference

[d] Current smoker

[e] General secondary school or lower

[f] HEI below the median of the analysed population

[g] TALIA below the median of the analysed population

[h] PHQ-4 score of ≥ 3 points

Table S6 Associations between sociodemographic, health and lifestyle factors and the odds of iatrogenic preterm birth – models including linear covariates

Covariate	Model 1	Model 2	Model 3	Model 4
**Group assignment**	0.64 (0.32 – 1.27)	0.63 (0.31 – 1.25)	0.62 (0.31 – 1.25)	0.64 (0.32 – 1.27)
**BMI**[a]	0.98 (0.91 – 1.06)	0.98 (0.91 – 1.06)	0.98 (0.91 – 1.06)	0.98 (0.90 – 1.06)
**Age**	**1.11 (1.03 – 1.20)**[+]	**1.11 (1.03 – 1.21)**[+]	**1.12 (1.03 – 1.21)**[+]	**1.12 (1.04 – 1.22)**[+]
**Parity**		0.90 (0.55 – 1.47)	0.88 (0.54 – 1.44)	0.91 (0.55 – 1.50)
**Early GWG**[b]		0.98 (0.84 – 1.13)	0.97 (0.84 – 1.13)	0.98 (0.84 – 1.13)
**Smoking**[c] **(categorial)**			2.24 (0.64 – 7.76)	1.98 (0.56 – 7.00)
**Low education**[d] **(categorial)**			1.01 (0.37 – 2.73)	0.95 (0.35 – 2.58)
**HEI score**[e]				0.81 (0.54 – 1.21)
**PA**[f]				1.00 (0.95 – 1.05)
**Antenatal distress**[g] **(categorial)**				1.22 (0.62 – 2.41)

BMI body mass index, *GWG* gestational weight gain, *HEI* Healthy Eating Index, *MET* metabolic equivalent of task, *PA* physical activity, *PHQ-4* Patient Health Questionnaire-4, *TALIA* total physical activity of light intensity and above

[+] $p < 0.05$

[a] Effect sizes are calculated per unit of measurement respectively

[b] GWG until the 16th – 20th week of gestation

[c] Current smoker

[d] General secondary school or lower

[e] Effect sizes are calculated per 10 HEI points

[f] TALIA, effect sizes are calculated per 10 MET-h/week

[g] PHQ-4 score of ≥ 3 points

Table S7 Associations between sociodemographic, health and lifestyle factors and the odds of spontaneous preterm birth – models including categorical covariates

Covariate	Model 1	Model 2	Model 3	Model 4
**Group assignment**	1.57 (0.98 – 2.52)	1.49 (0.93 – 2.40)	1.50 (0.94 – 2.42)	1.54 (0.96 – 2.48)
**BMI category**[a]				
BMI 25.0 – 29.9 kg/m^2	1.27 (0.75 – 2.16)	1.33 (0.78 – 2.27)	1.32 (0.77 – 2.26)	1.29 (0.75 – 2.21)
BMI 30.0 – 40.0 kg/m^2	1.07 (0.52 – 2.23)	1.05 (0.50 – 2.19)	1.04 (0.49 – 2.19)	0.99 (0.47 – 2.10)
**Age**[b]				
26 – 35 years	1.26 (0.59 – 2.68)	1.40 (0.65 – 3.00)	1.38 (0.64 – 2.96)	1.45 (0.67 – 3.14)
36 – 43 years	1.43 (0.56 – 3.63)	1.76 (0.68 – 4.55)	1.73 (0.66 – 4.48)	1.81 (0.69 – 4.74)
**Nulliparity**		**1.68 (1.01 – 2.78)**$^+$	**1.69 (1.02 – 2.80)**$^+$	1.63 (0.96 – 2.77)
**Early GWG**[c]				
Excessive		0.86 (0.42 – 1.76)	0.88 (0.43 – 1.80)	0.87 (0.43 – 1.77)
Inadequate		1.07 (0.49 – 2.32)	1.09 (0.50 – 2.36)	1.03 (0.48 – 2.24)
**Smoking**[d]			0.24 (0.03 – 1.78)	0.23 (0.03 – 1.67)
**Low education**[e]			1.31 (0.69 – 2.51)	1.25 (0.65 – 2.40)
**Low HEI**[f]				1.48 (0.92 – 2.39)
**Low PA**[g]				1.03 (0.64 – 1.67)
**Antenatal anxiety/ distress**[h]				0.93 (0.58 – 1.49)

BMI body mass index, *GWG* gestational weight gain, *HEI* Healthy Eating Index, *IOM* Institute of Medicine, *PA* physical activity, *PHQ-4* Patient Health Questionnaire-4, *TALIA* total physical activity of light intensity and above

$^+$ $p < 0.05$

[a] BMI 18.5 – 24.9 kg/m^2 was used as reference

[b] Age 18 – 25 years was used as reference

[c] GWG until the 16th – 20th week of gestation, defined according to the criteria of the IOM, adequate GWG was used as reference

[d] Current smoker

[e] General secondary school or lower

[f] HEI below the median of the analysed population

[g] TALIA below the median of the analysed population

[h] PHQ-4 score of ≥ 3 points

Table S8 Associations between sociodemographic, health and lifestyle factors and the odds of spontaneous preterm birth – models including linear covariates

Covariate	Model 1	Model 2	Model 3	Model 4
**Group assignment**	1.59 (0.99 – 2.55)	1.51 (0.94 – 2.43)	1.53 (0.95 – 2.45)	1.54 (0.96 – 2.47)
**BMI**[a]	1.01 (0.96 – 1.07)	1.01 (0.96 – 1.06)	1.01 (0.96 – 1.06)	1.00 (0.95 – 1.06)
**Age**	1.00 (0.95 – 1.06)	1.02 (0.97 – 1.08)	1.02 (0.96 – 1.07)	1.02 (0.96 – 1.07)
**Parity**		0.70 (0.47 – 1.04)	0.69 (0.47 – 1.04)	0.75 (0.50 – 1.14)
**Early GWG**[b]		0.95 (0.86 – 1.05)	0.95 (0.86 – 1.05)	0.95 (0.86 – 1.05)
**Smoking**[c] **(categorial)**			0.25 (0.03 – 1.80)	0.24 (0.03 – 1.77)
**Low education**[d] **(categorial)**			1.32 (0.69 – 2.53)	1.33 (0.69 – 2.56)
**HEI score**[e]				0.94 (0.71 – 1.23)
**PA**[f]				0.98 (0.94 – 1.01)
**Antenatal distress**[g] **(categorial)**				0.92 (0.58 – 1.48)

BMI body mass index, *GWG* gestational weight gain, *HEI* Healthy Eating Index, *MET* metabolic equivalent of task, *PA* physical activity, *PHQ-4* Patient Health Questionnaire-4, *TALIA* total physical activity of light intensity and above

[+] $p < 0.05$

[a] Effect sizes are calculated per unit of measurement respectively

[b] GWG until the 16th – 20th week of gestation

[c] Current smoker

[d] General secondary school or lower

[e] Effect sizes are calculated per 10 HEI points

[f] TALIA, effect sizes are calculated per 10 MET-h/week

[g] PHQ-4 score of ≥ 3 point
